# Quantitative data collection approaches in subject-reported oral health research: a scoping review

**DOI:** 10.1186/s12903-022-02399-5

**Published:** 2022-10-03

**Authors:** Carl A. Maida, Di Xiong, Marvin Marcus, Linyu Zhou, Yilan Huang, Yuetong Lyu, Jie Shen, Antonia Osuna-Garcia, Honghu Liu

**Affiliations:** 1 Division of Oral and Systemic Health Sciences, School of Dentistry, University of California, Los Angeles, 10833 Le Conte Ave, Los Angeles, CA USA; 2 Department of Biostatistics, Fielding School of Public Health, University of California, Los Angeles, 650 Charles E Young Drive South, Los Angeles, CA USA; 3 Louise M. Darling Biomedical Library, University of California, Los Angeles, 12-077 Center for Health Sciences, Los Angeles, CA USA; 4 Division of General Internal Medicine and Health Services Research, Geffen School of Medicine, University of California, Los Angeles, 10833 Le Conte Ave, Los Angeles, CA USA

**Keywords:** Quantitative research, Patient-reported outcomes, Dental disease experience, Oral health-related quality of life, Data collection

## Abstract

**Background:**

This scoping review reports on studies that collect survey data using quantitative research to measure self-reported oral health status outcome measures. The objective of this review is to categorize measures used to evaluate self-reported oral health status and oral health quality of life used in surveys of general populations.

**Methods:**

The review is guided by the Preferred Reporting Items for Systematic Reviews and Meta-Analyses Extension for Scoping Reviews (PRISMA-ScR) with the search on four online bibliographic databases. The criteria include (1) peer-reviewed articles, (2) papers published between 2011 and 2021, (3) only studies using quantitative methods, and (4) containing outcome measures of self-assessed oral health status, and/or oral health-related quality of life. All survey data collection methods are assessed and papers whose methods employ newer technological approaches are also identified.

**Results:**

Of the 2981 unduplicated papers, 239 meet the eligibility criteria. Half of the papers use impact scores such as the OHIP-14; 10% use functional measures, such as the GOHAI, and 26% use two or more measures while 8% use rating scales of oral health status. The review identifies four data collection methods: in-person, mail-in, Internet-based, and telephone surveys. Most (86%) employ in-person surveys, and 39% are conducted in Asia-Pacific and Middle East countries with 8% in North America. Sixty-six percent of the studies recruit participants directly from clinics and schools, where the surveys were carried out. The top three sampling methods are convenience sampling (52%), simple random sampling (12%), and stratified sampling (12%). Among the four data collection methods, in-person surveys have the highest response rate (91%), while the lowest response rate occurs in Internet-based surveys (37%). Telephone surveys are used to cover a wider population compared to other data collection methods. There are two noteworthy approaches: 1) sample selection where researchers employ different platforms to access subjects, and 2) mode of interaction with subjects, with the use of computers to collect self-reported data.

**Conclusion:**

The study provides an assessment of oral health outcome measures, including subject-reported oral health status and notes newly emerging computer technological approaches recently used in surveys conducted on general populations. These newer applications, though rarely used, hold promise for both researchers and the various populations that use or need oral health care.

**Supplementary Information:**

The online version contains supplementary material available at 10.1186/s12903-022-02399-5.

## Background

A fundamentally different approach is currently needed to address the oral health of populations worldwide namely by considering the perspective of patients or populations and not only dental professionals' views [1]. It seems increasingly necessary to integrate the self-reported perceptions of oral health, as they can complete or even replace clinical measures of dental status in surveys of populations. Indeed, such subjective measures are easy to use in large-scale populations and can provide a broader health perspective as compared to clinically determined measures of dental status alone [2, 3]. Since the topic is broad, this scoping review sets out to identify methods employed in population surveys that discussed self-reported perceptions of oral health, and the extent to which new computer-oriented technological approaches are being incorporated in the research methods.

The literature on oral health and dental-related scoping and systematic reviews includes studies that use specific populations in terms of disease or clinical conditions, treatments, political or social status and typically do not explore oral health status outcome measures [4–15]. These studies only occasionally provide perspectives on general populations. A review by Mittal et al. identifies dental Patient-Reported Outcomes (dPROs), and dental Patient Reported Outcome Measures (dPROMs) related to oral function, oral-facial pain, orofacial pain and psychosocial impact [16]. The study affords a valuable and extensive review of self-reported oral health and quality of life measures, many of which are found in this paper. This scoping review, then, seeks approaches used in subject-reported surveys, including those with general populations, which may broaden the perspective on oral health outcome measures.

The objective of this review is to categorize measures used to evaluate self-reported oral health status and oral health quality of life used in surveys of general populations.

## Methods

This work is implemented following the framework of scoping reviews [17–19] and is presented according to the recommendations of the Preferred Reporting of Items for Systematic Reviews and Meta-Analyses Extension for Scoping Reviews (PRISMA-ScR), as listed in Additional file 1: Preferred Reporting Items for Systematic reviews and Meta-Analyses extension for Scoping Reviews (PRISMA-ScR) Checklist [20]. Additional file 5: Glossary of Terms provides definitions for the important terms used across the paper.

### Search strategy and data sources

A health science librarian assisted in the development of a search strategy that identified papers concerning subject-reported oral health status surveys. The search terms consisted of three broad categories, including survey methods, subject-reported outcomes, and oral health and disease (see Additional file 2: Search Terms for the full list of search strings). The search comprised peer-reviewed journal articles, conference proceedings and reviews with at least one keyword from each of three aspects. Four online databases: Ovid Medline, Embase, Web of Science, and Cochrane Reviews and Trials were used. In addition, a manual search used similar keywords for the gray literature achieved on MedRxiv. The search focused on peer-reviewed papers written in English and published between 2011 to September 2021. Publications in the last decade were reviewed to investigate the extent to which different methods were being used and the trends that occurred during this period. The final search was completed on September 29, 2021. Using the current decade provides a period where there is considerable interest in non-clinical oral health status outcome measures and the potential for examining technological innovation. All references were imported for review and appraisal. Duplicates were identified using Mendeley (Mendeley, London, UK) and manually verified. After removing the duplicates, data were tabulated in Microsoft Excel (Microsoft, Redmond, WA, USA) for recording screening results and data charting.

### Study inclusion criteria

Studies that did not meet with the research objective were excluded using a screening tool (Additional file 3: Search Tool). First, the titles and abstracts of publications were screened to determine if studies conducted quantitative surveys, and to assess if self-reported and/or proxy-reported OHS was a primary objective. Only surveys with more than three questions that related to OHS were considered. Studies with secondary analysis were excluded because the data collection methods were normally not developed as part of the research and were developed previously. Papers whose sole purpose was to validate well-known measures of oral health were also rejected since the intent was not to assess the OHS of a population. Literature reviews were likewise excluded, as were papers describing results from focus groups and other qualitative studies. Papers whose objectives were to validate measures or predict specific oral disease entities, such as caries or gingival bleeding, rather than overall OHS. Studies primarily focusing on general health status or other systemic diseases instead of OHS were eliminated. Randomized Controlled Trials (RCT) or quasi RCT studies that tested an active agent (e.g., therapy, experiment, and medicine) were excluded because the main research purpose was a comparison of treatment rather than an assessment of subject-reported OHS.

The research team performed the secondary screening through a full-text review. We dropped papers with full text missing or not in English. Then, we screened the available full-text works using a similar set of inclusion criteria aforementioned and further excluded papers without information about data collection methods.

### Selection strategies

**Fig. 1:**
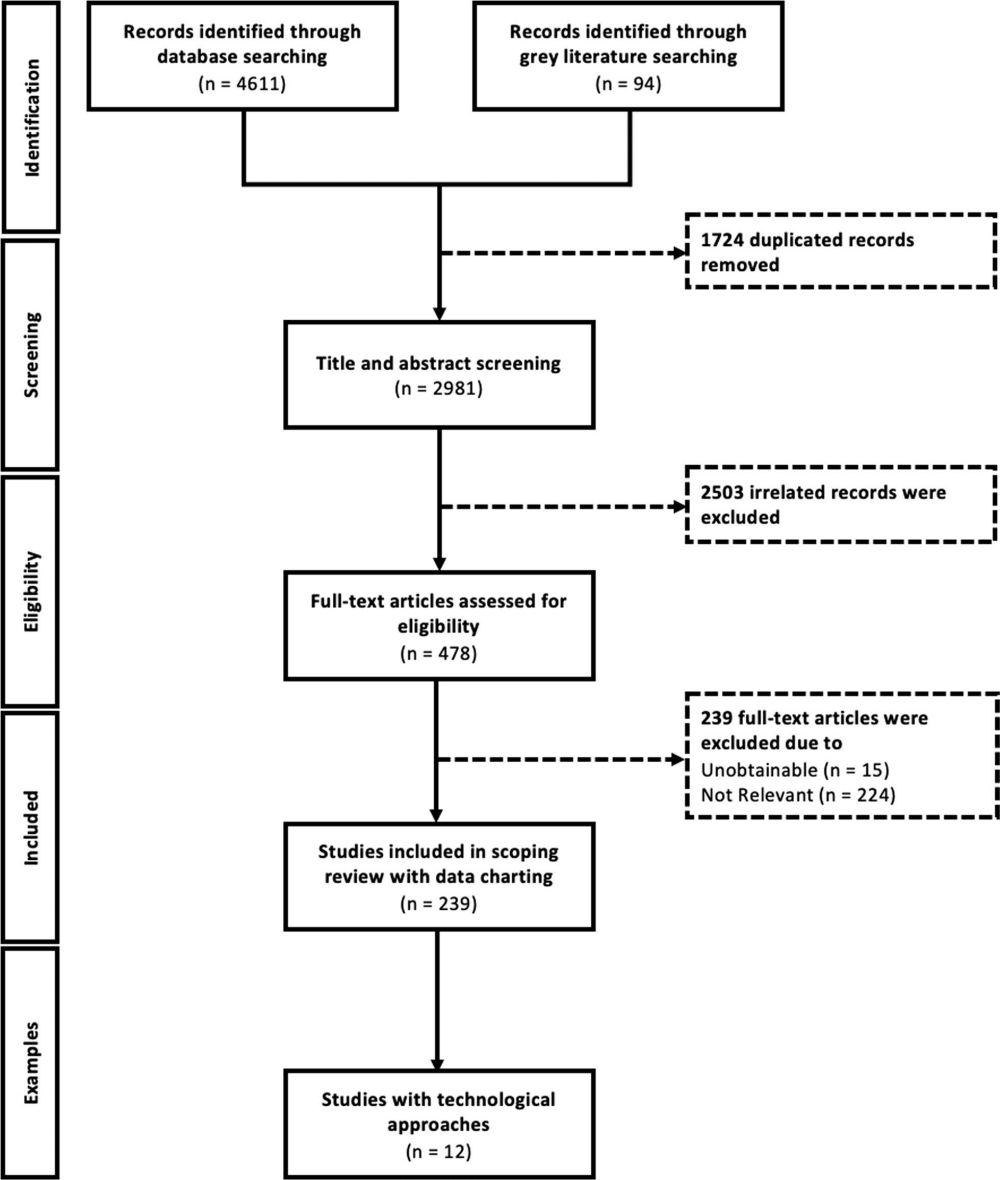
PRISMA framework with additional examples

Figure 1 outlines the review process utilizing the PRISMA-ScR framework. The title-and-abstract screening was completed by a researcher (D.X.) against the inclusion criteria using a screening tool (Additional file 3: Search Tool). To check for reliability and consistency, one of the researchers (L.Z.) randomly screened 10% of articles independently and compared the inclusion decisions. Given the result of title-and-abstract screening, two researchers (L.Z. and Y.H.) verified the eligibility of the remaining articles independently through full-text review. Inclusion discrepancies were resolved by an additional researcher (D.X.).

### Data extraction

The data charting form (Additional file 4: Data Charting Form) consists of quantitative and qualitative variables for the data collection methods and their characteristics, such as outcome measures, use of assistive devices/tools or data sources, report type, and so on. The form has been pre-tested by two project staff (C.M. and M.M.) before being utilized. Two researchers (Y.H. and L.Z.) extracted data using the form. Two project staff (C.M. and M.M.) collaborated to review the charted study characteristics and the discrepancies have been addressed through discussion.

### Data synthesis and analysis

The scoping review synthesizes the research findings based on dimensions and attributes of major oral health survey data collection methods using descriptive and content analyses. The review provides an overview of various related data collection methods in the recent literature, which refer to the quantitative methods to collect information from a pool of respondents, and the trends in using these new technological approaches, which involve computerized modes, Internet-supported devices and interactive web technologies. Through the literature review, we locate four major types of data collection methods: in-person, Internet-based, telephone-based, and mail-in based approaches.

## Results

### Screening and study selection

After removing duplicates, the initial search revealed 2981 articles from four online databases for title-and-abstract screening; 2503 of which were excluded after being examined against the inclusion criteria. The interrater reliability of screening was measured by Kappa agreement as 0.94 (95% confidence interval [0.89, 0.99]) for title-and-abstract screening, which implies almost perfect agreement [21]. After full-text reviewing and excluding 239 articles, we summarized and categorized the remaining 239 studies based on the pre-tested data charting form. In addition, we identified 12 studies with various technological approaches to data collection. Figure 1 presented the PRISMA Framework used for this scoping review.

### General characteristics of included studies

Table 1 presents various characteristics of the 239 articles that meet inclusion criteria that were published from 2011 to September 2021. Fifty-six percent of the papers are published in dental journals. About 40% of the papers are published in journals from the Asia-Pacific and Middle East region (APAC), and only 8.4% are from North America (NA). The majority of studies (69%) focus on the general population. Most (88.6%) of the studies use in-person surveys. Around two-thirds of the studies invite and recruit participants from the study sites, e.g., schools, clinics, and hospitals. Some studies recruit participants by having the research team visit communities (16%) or by sampling directly from a database (13%). In the latter case, participants are selected using probability and/or non-probability sampling methods, including convenience sampling (52%), simple random sampling (12%), and stratified sampling (12%). Most studies (193 or 80.8%) investigate self-reported outcomes. Dental examinations accompany the survey in 54% of the studies, while 32% of studies do not use any clinical exam or records. The data charting details are listed in Additional file 4: Data Charting Form.

**Table 1:** Characteristics of studies analyzed

	Number of studies (n)	Percentage (%)
**Type of study**		
Peer-reviewed Articles	238	99.6
Preprint Papers	1	0.4
**Publication year**		
2011–2012	32	13.4
2013–2014	42	17.6
2015–2016	39	16.3
2017–2018	52	21.8
2019–2020	50	20.9
2021 (up to September)	24	10.0
**Journal type**		
Dental Journal	132	55.2
Non-Dental Journal	107	44.8
**Region**		
APAC: Asia/Pacific (including the Middle East)	94	39.3
EUR: Europe	59	24.7
LATAM: Latin America	56	23.4
NA: North America	20	8.4
AFR: Africa	10	4.2
**Data collection start year**		
Before–2005	3	1.3
2006–2010	55	23.0
2011–2015	62	25.9
2016–2020	50	20.9
Missing	69	28.9
**Data collection methods**		
In-Person	206	86.2
Mail-in	15	6.3
Internet-based	6	2.5
Telephone	3	1.3
Mixed	9	3.8
**State of health**		
General health	163	68.2
Medical problem	47	19.7
Dental problem	29	12.1
**Recruitment sources**		
Direct recruitment	157	65.7
Community-based	39	16.3
Database	30	12.6
Hardcopy advertisements	4	1.7
Web-based advertisements	1	0.4
Mixed	7	2.9
Others	1	0.4
**Sampling methods**^1^		
Convenience sampling [NP]	124	51.9
Simple random sampling [P]	29	12.1
Stratified sampling [P]	28	11.7
Cluster sampling [P]	21	8.8
Purposive sampling [NP]	8	3.3
Consecutive sampling [NP]	7	2.9
Systematic sampling [P]	6	2.5
Snowball sampling [NP]	3	1.3
Mixed (more than 1 method used)	9	3.8
Other/no sampling methods	4	1.7
**Report type**		
Self-reported	193	80.8
Proxy	19	7.9
Both	27	11.3
**Use of assistive devices/tools or data sources**		
Clinical dental exam	129	54.0
Medical or dental records	9	3.8
Public database (national surveys)	3	1.3
Mixed: exams and records	12	5.0
Mixed: exams and public database	2	0.8
Others	8	3.3
None	76	31.8

^1^[NP] non-probability sampling method; [P] probability sampling method

### Characteristics of data collection methods

The four main data collection methods include in-person (N = 206, 86.2%), mail-in (N = 15, 6.3%), Internet-based (N = 6, 2.5%), and telephone-based (N = 3, 1.3%) surveys. The characteristics of the various data collection methods are summarized in Table 2.

**Table 2:** Dimensions and attributes of various data collection methods

Dimensions	Data collection methods (number of papers, %)			
	In-person (N = 206, 86.2%)	Mail-in (N = 15, 6.3%)	Internet-based (N = 6, 2.5%)	Telephone (N = 3, 1.3%)
**Technological approaches**^1^	Face-to-face interview (55.8%)	Via Post (80.0%)	Computer-Assisted Web Interview (CAWI) and Online Survey (66.7%)	Interview (100%)
	Paper and Pencil (35.4%)	By Carrier (e.g., sent home with the child) (20.0%)	Email (33.3%)	
	Mixed (6.4%)			
	Computer-Assisted Personal Interview (CAPI) and Electronic Survey (1.9%)			
	Uncertain (0.5%)			
**Recruit sources**^1^	Direct Recruitment (69.4%)	Direct Recruitment (33.3%)	Mixed (50.0%)	Database (66.7%)
	Community-based (17.5%)	Database (33.3%)	Direct Recruitment (16.7%)	Direct Recruitment (33.3%)
	Database (9.7%)	Community-based (20.0%)	Database (16.7%)	
	Mixed (1.5%)	Hard-copy Advertisement (6.7%)	Web-based (16.7%)	
	Hard-copy Advertisement (1.5%)	Mixed (6.7%)		
	Others (0.5%)			
**Sampling methods**^1,2^	Convenience Sampling [NP] (54.4%)	Simple Random Sampling [P] (46.7%)	Convenience Sampling [NP] 66.7%)	Simple Random Sampling [P] (33.3%)
	Stratified Sampling [P] (10.7%)	Stratified Sampling [P] (20.0%)	Snowball Sampling [NP] (33.3%)	Stratified Sampling [P] (33.3%)
	Simple Random Sampling [P] (9.7%)	Convenience Sampling [NP] (20.0%)		Convenience Sampling [NP] (33.3%)
	Cluster Sampling [P] (9.7%)	Purposive Sampling [NP] (6.7%)		
	Purposive Sampling [NP] (3.4%)	Others (6.7%)		
	Consecutive Sampling [NP] (3.4%)			
	Systematic Sampling [P] (2.9%)			
	Snowball Sampling [NP] (0.5%)			
	Mixed (4.4%)			
	Others (1.0%)			
**Duration length of data collection, median (Min, Max)**	1 (0, 12) in years	0 (0, 1) in years	0 (0, 1) in years	0.5 (0, 1) in years
**Sample size, median**	321	879	259	1500
**Response rate (unadjusted), median**	90.6%	72%	36.7%	55.5%
**Use of assistive devices/tools or data sources**^1^	Clinical Dental Exam (58.3%)	Clinical Dental Exam (26.7%)	None (100.0%)	None (100.0%)
	Public Database (5.3%)	Medical or Dental Records (6.7%)		
	Medical or Dental Records (3.9%)	None (66.6%)		
	Mixed: Exams and Public Database (1.5%)			
	Mixed: Exams and Records (0.9%)			
	Others (2.9%)			
	None (27.2%)			

^1^Re-ordered by the percentage of studies in each category

^2^[NP] non-probability sampling method; [P] probability sampling method

The majority of the studies using in-person surveys have high response rates with an average of 90.6%. Those studies using in-person survey methods represent half 55.8% of the studies employ face-to-face interviews, while 35.4% used a paper-and-pencil approach. Participants for 58.7% of the studies are recruited directly from clinics [22], hospitals [23], and community care centers [24]. For those sites with electronic records, additional data sources are directly linked to the survey, for example, clinical dental exams with visual components (e.g., X-ray [25] and pictures [26]) and medical records [23, 27, 28]. Moreover, different qualitative assessments (e.g., Malocclusion Assessment [22] and Masticatory Performance Test [24, 29]) are captured in patient progress notes.

The mail-in survey method is used by 15 studies and may be more cost-effective than in-person delivery, though these were the two main sources, via post (80%) and by carriers (20%). Mail-in surveys have a relatively high response rate averaging 72%, especially when children or other respondents bring surveys home to complete. Similar to in-person surveys, mail-in surveys can incorporate additional resources, such as photographs and explanations of clinical conditions and treatments [30, 31].

Only six studies are identified as using an Internet-based survey, mainly through computer-assisted web interviews (4 studies), and email (2 studies). Three papers employ direct recruitment and another three papers recruit participants through websites and databases. The average response rate is as low as 36.7% for this method with small sample sizes with a median of 259 participants.

Three studies use a telephone survey method covering large populations compared to other survey methods with more responders on average. Two of these studies recruit participants through an existing database, and all surveys used interviewers. Computer-Assisted Telephone Interviews (CATI) [32] and Voice Response Systems [33] which are commonly used in industry are not found in the studies.

In addition to the data collection methods, we further categorize the measures found in the 239 articles. Table 3 presents the frequencies and percentages of the various self-reported outcome measures. The three basic approaches are oral health impact measures [34], functional measures [34], and self- or proxy-ratings of OHS, with the terms defined in Additional file 5: Glossary of Terms. These are used as single measures or in combination. The Oral Health Impact Profile-14 (OHIP-14) is the most prevalent single measure with 69 papers and 29% overall, of which 25 papers are about child impact, representing 10% of the total number of selected papers. The Geriatric Oral Health Assessment Index (GOHAI), a functional measure, is second with 21 papers and 9% overall. The GOHAI is the first among the studies on the elderly. There are also two adolescent papers representing 9% of the functioning category. The self- or proxy-rating of OHS has 18 single-measure papers representing 8% of these articles. Of these, 12 or 80% are children's measure's, representing 5% of all selected papers.

**Table 3:** Number and percent of oral health-related quality of life and self-reported oral health status by type, group and combinations

Major characteristic	Description	Measures	Number of studies	Percent by group (%)	Percent Overall (%)
**Impact measures**	Evaluate impacts caused by functional limitations, physical, psychological and social factors	Oral Health Impact Profile 14 (OHIP-14) [35]	69	58	29
		Oral Impacts on Daily Performances (OIDP) [36]	20	17	8
		Early Childhood Oral Health Impact Scale (ECOHIS) [37]	15	13	6
		Child Oral Impacts on Daily Performance (C-OIDP) [38]	7	6	3
		Oral Health Impact Profile (OHIP-49) [39]	3	3	1
		Child Oral Health Impact Profile (COHIP) [40]	3	3	1
		Other Impact Measures	3	3	1
		**Total**	120	100	50
**Functioning measures**	Assess physical, social and psychosocial functions of the elderly	Geriatric Oral Health Assessment Index (GOHAI) [41]	21	91	9
	Assess physical, social, role, emotional, and oral problems	Teen Oral Health-related Quality of Life (TOQOL) [42]	2	9	1
		**Total**	23	100	10
**Perceived OHS and functioning measures**	Evaluate perceived oral health status score, functional limitations, emotional and social well-being	Child Perceptions Questionnaire 8–10 (CPQ8–10) [43]	1	7	0
		Child Perceptions Questionnaire 11–14 (CPQ11–14) [44]	11	73	5
		Multiple CPQ	3	20	1
		**Total**	15	100	6
**Self-rating of OHS measures (only)**		Self-rating of OHS (only)	18	100	8
		**Total**	18	100	8
**Using more than one mea﻿sure**		More than one measure is used (excluding self-rating scales)	36	100	15
		Combination of self-rating scale and oral health quality of life measure	27	100	11
		**Total**	239	100	100

There is a total of 63 papers using more than one type of measure. Either combining functional and impact measures (36 and 15%) or self-rating OHS and one or more of the other measures (27 or 11%). The group of single impact measures is 50% of the overall and also represents where two or more measures were used. The single measure, GOHAI, based on function is only 9% of all measures but also played a role in combination with other measures. Finally, the self-reported OHS as a single measure represents 8% of the studies. Its role is mainly in combination with other measures and represented another 15% of the articles. In total. children's oral health measures form a considerable portion of the self-reported oral health outcome research papers, representing 16% of all studies. There are additional studies where children’s measures are used in combination with adults.

Currently, the use of technological approaches emerged in the field of survey research to improve the quality and quantity of data collection. After reviewing and charting all qualified 239 articles, twelve studies that employ technological approaches are summarized in Table 4.

**Table 4:** Summary of measures, and data collection methods and technological approaches for the selected studies (N = 12, ordered by data collection methods and year)

Study (author-year-country)	Study population	Sample size (response rate, %)	Sampling methods	Data collection methods and technological approaches	Measures
Broughton et al.-2012-New Zealand [45]	Healthy Māori teenagers (16–18) in New Zealand	238 (NA)	Convenience sampling on Rangatahi as undertaken in the Rohe of Tainui	In-Person CAPI and electronic survey under the supervision of two specifically trained Māori health research assistants	Self-reported oral health (OHIP-14) and use of oral health services
Liu et al.-2018-US [2]	Patients (age 8–17) without orthodontic appliances, and their parents or guardians, reflecting the general US population	334 (NA)	Convenience sampling with clinic-based recruitment of patients and their parents/guardians at dental clinics located in LA County	In-person CAPI and electronic survey using Questionnaire Development System™ (QDS™, NOVA Research Company, Silver Spring, MD, USA)	Self- and proxy-reported oral health status of children and adolescents
Morgan et al.-2018-Rwanda [46]	Individuals representing the general population of Rwanda	2097 (NA)	Random sampling using pathfinder methodology which is a stratified cluster sampling technique aiming to include the most important population subgroups likely to have differing disease levels	In-person CAPI and an electronic survey administered by local community leaders at the district	Self-reported oral health practices, behaviors, and related quality of life
Echeverria et al.-2020-Brazil [47]	Healthy college students in Brazil	1865 (69%)	Convenience sampling of university students	In-person CAPI and electronic survey using RedCap (Vanderbilt University, Nashville, Tennessee, USA) installed on tablets	Self-rating on oral health status and use of dental services
Mohamad Fuad et al. 2020—Malaysia [48]	Older persons in Malaysia (age > 60)	3867 (97.2%)	Stratified cluster sampling strategy with primary stratum constitutes the states and federal Living Quarters (LQs) selections, followed by the secondary stratum (urban and rural areas)	In-person face-to-face interview using tablets	Self-reported OHRQoL by older persons (GOHAI Malay version), and self-perception of oral health
Hanisch et al.-2018—Germany [49]	German patients affected by a rare disease (age > 16)	451 (NA)	The questionnaire in electronic file format was sent digitally to the Alliance of Chronic Rare Diseases (ACHSE e.V.). Snowball sampling participants with the help of participants on recruiting other participants for the study	Internet-based online survey	Self-reported OHRQoL (OHIP-14), free text questions addressing participants' satisfaction with the dental treatment and the health care system
Nam et al.-2017-Korea [50]	Healthy university students in Korea (age > = 20)	130 (NA)	A random sampling of students in 3 majors at Kangwon University Dogye Campus	An Internet-based online survey using Google Forms (Google LLC., Mountain View, CA, USA)	Self-reported quality of life, dental health status, and education
Mortimer-Jones et al.-2018-Australia [51]	Australian healthy nursing students across all year levels	281 (25%)	Snowball sampling that study info was circulated by staff members and publicized during lectures. Convenience sampling by recruiting all nursing students in email form	An Internet-based online survey using SurveyMonkey (Momentive Inc., San Mateo, CA, USA)	Self-reported anxiety and temporomandibular-related symptoms, and quality of life (OHIP-TMD and PROMIS® short form)
Liu-2021-China [52]	Chinese Healthy Kids (3–6)	4495 (NA)	Convenience sampling with online recruitment using a recruitment link or quick response (QR) code to be distributed to groups in their WeChat	Internet-based survey platform	Proxy-reported oral health status, care behavior, and caregivers’ attitudes
			Snowball sampling that people can recruit other participants by sharing the link		
Makizodila-2021-Netherlands [53]	Motor Neuron Disease (MND) patients in Netherland	259 (36.7%)	Convenience sampling on caregivers of all registered MND patients in the Netherlands in the Prospective ALS study Netherlands Database by an email newsletter of the Dutch ALS Centre	Email	Self- and proxy-reported OHRQoL (GOHAI) and their performance on oral hygiene
Kotzer et al.-2012-Canada [54]	Canadian healthy pre-seniors and seniors	1461 (NA)	Facility-based as well as random sampling of pre-seniors and seniors	Telephone interviews for community residents and in-person face-to-face interviews for long-term care residents	Self-reported OHRQoL (OHIP-14), general health, and medication use
Hakeberg and Wide-2018-Sweden [55]	Swedish Healthy Adult Residents (age > 19)	3500 (49.7%)	Random sampling by a telemarketing company (TNS SIFO) selected participants from Swedish Personal Address Registry	Telephone interview	Self-reported Dental anxiety, health-related quality of life (OHIP-5 and EQ-5D) and several socioeconomic variables

*CAPI* Computer Assisted Personal Interviewing; *OHIP-14* Oral Health Impact Profile 14; *OHRQoL* Oral Health-related Quality of Life; *GOHAI* Geriatric Oral Health Assessment Index; *PROMIS* Patient-Reported Outcomes Measurement Information System; *ALS* Amyotrophic Lateral Sclerosis; *EQ-5D* EuroQol-5D

## Discussion

This scoping review provides an overview of data collection methods used for subject-reported surveys to measure oral health outcomes. Studies are characterized by four survey methods (in-person, mail-in, Internet-based, and telephone) and by summarized dimensions and attributes of data collection for each method, such as technological approaches, survey population or sampling methods. Studies typically employ in-person surveys and more studies were conducted in Asia-Pacific and Middle East countries than in any other world region. Most studies recruit participants directly from study sites. Both probability and non-probability sampling methods employ typically convenience sampling, simple random sampling, and stratified sampling. Studies that achieve the highest response rate on average use in-person surveys, while the lowest rate occurs in Internet-based surveys. Telephone surveys are used to cover a wider population compared to other data collection methods. Many studies, especially those using in-person and mail-in data collection methods, incorporate supplemental data types and technological approaches. Outcome measures are frequently used to evaluate impacts caused by functional limitations related to physical, psychological, and social factors.

Frequently used self-reported oral health status and OHRQoL measures are OHIP-14, an impact measure, and the GOHAI, a functional measure. Children’s oral health outcomes measures form a considerable portion of the self-reported oral health outcome research papers. Although OHIP-14 is the most utilized single measure, many other papers use only portions of this measure, while adding other outcome measures, such as dental care needs, satisfaction, oral health status, and so on. The validity of these measures is therefore compromised and could not provide insight into the degree that the studies are measuring self-reported oral health status or quality of life [4]. Other measures rate an individual’s oral health status using a simple self-rating scale, from very poor to excellent. This approach is more directly related to a person’s oral conditions and therefore their perceptions and behavior tend to be more consistent with this rating [56, 57]. These self-rating measures focus on the overall dimension of perceived oral health status. Unlike the measures previously discussed, these simple ratings do not delineate the psychological, social and physical dimensions of oral health. Nevertheless, such measures can enable researchers to identify hidden dimensions by analyzing independent variables that account for the respondent’s perception.

This review identifies research that employs more conventional methods. The face-to-face interview and the pencil and paper format are conventionally used in many studies along with a clinical dental exam. While offering unique flexibility and easier administration, in-person approaches are more labor-intensive and normally take more time compared to other methods. Countries, such as Brazil, rely for years on these techniques to develop national epidemiological oral health surveys [28, 58, 59]. Although these surveys are very well-organized and established throughout the country, this review does not find that newer technological approaches are introduced into their conventional approach. In this case, there may be little incentive to change their approach because their methods are well understood and employing more technological approaches may be costly.

The use of Internet-based surveys is increasingly common in the medical field. Although these surveys end with potentially lower response rates, this approach is normally more cost-effective [60]. Internet-based surveys have many notable advantages, including easy administration, fast data collection process, lower cost, wider population coverage and better data quality with fewer overall data errors and fewer missing items [61–63]. However, this data collection method is constrained by sample bias, topic salience, data security concerns and low digital literacy that may affect response rates [62]. In settings where Internet-based surveys are not practical, longstanding and effective conventional oral health data collection methods in research will continue. It is evident from this review that the use of computerized technological approaches is limited. While such approaches in survey research improve the quality and quantity of data collection, only twelve studies in this review employ them. The most widely used technical approaches are Computer-Assisted Personal Interviewing (CAPI) and online survey platforms (e.g., Google Forms and SurveyMonkey).

Two noteworthy approaches to survey research methodology emerge from this review, particularly in: (1) sample selection, and (2) mode of interaction with research subjects. North American researchers found different platforms to access subjects for their studies. Canadian studies use random digit dialing to recruit and conduct computer-assisted interviews [54]. In the United States, researchers access existing polling populations or use Amazon’s MTurk platform for “workers” who are paid small amounts for each survey they respond to [64]. The second approach is the use of computers to collect self-reported data. The basic surveying technique is CAPI with interviewers directly entering the data into a database. There is also Computer-Assisted Telephone Interviewing (CATI), a survey technique, where the interviewer follows a scripted interview guided by a questionnaire that appears on the screen. A third Internet-based survey technique, the Computer-Assisted Web Interviewing (CAWI), requires no live interviewer. Instead, the respondent follows a script made in a program for designing web interviews that may include images, audio and video clips, and web-based information.

An innovative technological approach worth noting is the use of OralCam to perform self-examination using a smartphone camera [65]. The study applies research used in medicine to detect liver problems from face photos as well as other diseases [66]. The paper describes the use of a smartphone camera to interact with a computer using diagnostic algorithms, such as the deep convolutional neural network-based multitask learning approach. Based on over three thousand intraoral photos, the system learns to analyze teeth and gingiva. The smartphone camera takes a picture using a mouth opener. The computer’s algorithms analyze the captured picture, along with survey data, to diagnose several dental conditions including caries, chronic gingival inflammation, and dental calculus. This use of multitask learning technology, with the extensive availability of cell phones, may revolutionize oral health research and care.

This scoping review is limited to oral health survey-based studies in peer-reviewed journals and MedRxiv published in English between 2011 to 2021. A further limitation is that many of the reviewed papers do not adequately describe the methods they use to collect data. Publications using secondary data from national studies are excluded, The exclusion is based on the fact that these researchers are not engaged in designing the methods or conducting the data collection. Often, the publications refer to the original study to describe the method used. Also, the original data collection may have occurred before the time frame of this review. The fifteen papers that use secondary data published over this study’s time frame represent only about six percent of the reviewed papers. Thus, the overall impact of this exclusion is minimal on this scoping review’s results.

## Conclusions

This scoping review provides an assessment of oral health outcome measures, including subject-reported oral health status, and notes newly emerging computer technological approaches recently used in surveys conducted on general populations. Such technological approaches, although rarely used in the reviewed studies, hold promise for both researchers and the various populations that use or need oral health care. Future studies employing more developed computer applications for survey research to boost recruitment and participation of study subjects with wide and diverse backgrounds from almost unlimited geographic areas can then provide a broader perspective on oral health survey methods and outcomes.

## Supplementary Information

**Additional file 1.** Preferred Reporting Items for Systematic reviews and Meta-Analyses extension for Scoping Reviews (PRISMA-ScR) Checklist.

**Additional file 2.** Search Terms.

**Additional file 3.** Search Tool.

**Additional file 4.** Data Charting Form.

**Additional file 5.** Glossary of Terms.

## Data Availability

All data generated or analyzed during this study are included in this published article and its supplementary information files.

## Abbreviations

PRISMA-ScR: Preferred Reporting Items for Systematic Reviews and Meta-Analyses Extension for Scoping Reviews
APAC: Asia-Pacific (including the Middle East)
EUR: Europe
LATAM: Latin America
NA: North America
AFR: Africa
CAPI: Computer-Assisted Personal Interviewing
CAWI: Computer-Assisted Web Interviewing
CATI: Computer-Assisted Telephone Interview
OHRQoL: Oral Health-Related Quality of Life
OHIP-14: Oral Health Impact Profile-14
GOHAI: Geriatric Oral Health Assessment Index
OHS: Oral Health Status
