# Supplementary material for: Quantitative data collection approaches in subject-reported oral health research: a scoping review
Source: BMC Oral Health. 2022 Oct 3;22:435. doi: 10.1186/s12903-022-02399-5 (PMC9528129; doi:10.1186/s12903-022-02399-5)
Supplement: Supplementary file 2 — Additional file 2. Search Terms. [file 12903_2022_2399_MOESM2_ESM.docx]

**Appendix B – Search Terms**

Searching Date: 09/30/2021

Search Period: 01/01/2021-09/29/2021

The search terms cover:

1) Survey Modes

2) Subject-reported Outcomes

3) Oral Health and Diseases

| **Database** | **# of Papers** | **Literature Limitation** | **Search Code** |
| --- | --- | --- | --- |
| Ovid Medline | 956 | No limits for grey lit | Dental Health Surveys/ or "Surveys and Questionnaires"/ or ((survey or questionnaire) adj5 (method or modes or valid)).ti,ab. or survey.ti,ab. or ('In person' or interview or 'paper pencil' or 'computer assisted personal interview' or CAPI or 'electronic tablet' or 'internet based' or email or 'web survey' or 'computer assisted web interview' or CAWI or teledentistry or telephone or 'mobile interview').ti,ab. AND *"Quality of Life"/ or Patient Reported Outcome Measures/ or Patient Health Questionnaire/ or exp Self Report/ or ((patient or self or Subject) adj5 (report or measur)).ti,ab. AND *oral health/ or exp Dental Caries/ or ((oral or dental or tooth) adj5 (health or disease or caries)).ti,ab. |
| Embase | 1180 | Articles, Article in press, review, conference abstract, conference paper, conference review | Search strategy: 'dental disease assessment'/mj OR 'caries assessment'/mj OR 'questionnaire'/mj OR (((survey OR questionnaire) NEAR/5 (method* OR modes OR valid*)):ti,ab) OR survey:ti,ab OR 'in person':ti,ab OR interview:ti,ab OR 'paper pencil':ti,ab OR 'computer assisted personal interview':ti,ab OR capi:ti,ab OR 'electronic tablet':ti,ab OR 'internet based':ti,ab OR email:ti,ab OR 'web survey':ti,ab OR 'computer assisted web interview':ti,ab OR cawi:ti,ab OR teledentistry:ti,ab OR telephone:ti,ab OR 'mobile interview':ti,ab AND 'quality of life'/mj OR 'patient-reported outcome'/mj OR 'self report'/exp OR ((patient OR self OR subject) NEAR/5 (report* OR measur*)):ti,ab AND 'dental health'/mj OR 'dental caries'/exp OR ((oral OR dental OR tooth) NEAR/5 (health OR disease OR caries)):ti,ab |
| Web of Science | 2424 | Articles, Early Access, Review, proceedings papers, editorial materials | “Dental Health Surveys” or “Surveys and Questionnaires” or ((survey or questionnaire) NEAR/5 (method* or modes or valid*)) or survey or 'In person' or interview or 'paper pencil' or 'computer assisted personal interview' or CAPI or 'electronic tablet' or 'internet based' or email or 'web survey' or 'computer assisted web interview' or CAWI or teledentistry or telephone or 'mobile interview' AND "Quality of Life" or “Patient Reported Outcome Measures” or “Patient Health Questionnaire” or “Self Report” or ((patient or self or Subject) NEAR/5 (report* or measur*)) AND “oral health” or “Dental Caries” or ((oral or dental or tooth) NEAR/5 (health or disease or caries)) |
| Cochrane | 51 | No limits for publication types. | ID Search #1 MeSH descriptor: [Dental Health Surveys] this term only #2 MeSH descriptor: [Surveys and Questionnaires] this term only #3 (((survey or questionnaire) adj5 (method* or modes or valid*)) or survey or 'In person' or interview or 'paper pencil' or 'computer assisted personal interview' or CAPI or 'electronic tablet' or 'internet based' or email or 'web survey' or 'computer assisted web interview' or CAWI or teledentistry or telephone or 'mobile interview'):ti,ab,kw #4 #1 OR #2 OR #3 #5 MeSH descriptor: [Quality of Life] this term only #6 MeSH descriptor: [Patient Reported Outcome Measures] this term only #7 MeSH descriptor: [Patient Health Questionnaire] this term only #8 MeSH descriptor: [Self Report] explode all trees #9 (((patient or self or Subject) adj5 (report* or measur*))):ti,ab,kw #10 #5 OR #6 OR #7 OR #8 OR #9 #11 MeSH descriptor: [Oral Health] this term only #12 MeSH descriptor: [Dental Caries] explode all trees #13 (((oral or dental or tooth) adj5 (health or disease or caries))):ti,ab,kw #14 #11 OR #12 OR #13 #15 #4 AND #10 AND #14 |
