# Supplementary material for: Quantitative data collection approaches in subject-reported oral health research: a scoping review
Source: BMC Oral Health. 2022 Oct 3;22:435. doi: 10.1186/s12903-022-02399-5 (PMC9528129; doi:10.1186/s12903-022-02399-5)
Supplement: Supplementary file 3 — Additional file 3. Search Tool. [file 12903_2022_2399_MOESM3_ESM.docx]

**Appendix C - Search Tool**

**C.1. Title-and-abstract Screening Checking List**

Guiding Screening Question: Does the study include subject-reported surveys to measure oral health status?

Yes = Included

No = Excluded

To answer ‘Yes’ to the above guiding question, the study must meet all following criteria:

1. Does this study have a self-reported and/or proxy-reported survey to assess oral health status? Oral health status is a measure of overall condition of oral cavity, including concepts, such as oral quality of life, oral health impact, and subject rating of oral health.

Yes = move to the next criteria

No = exclude this study

1. Is this measure of oral health the primary objective of this study, even though other measures of general health or other diseases are included?

Yes = move to the next criteria

No = exclude this study

1. Is it primarily a quantitative survey research study?

Yes = move to the next criteria

No = exclude this study

1. Does the survey have more than 3 questions related oral health?

Yes = move to the next criteria

No = exclude this study

1. Does this study collect primary data to analyze?

Yes = move to the next criteria

No = exclude this study

1. Does this study use legacy measures to assess the oral health status of a population rather than merely validating of legacy measures of oral health?

Yes = move to the next criteria

No = exclude this study

1. Does this study focus on the self-reported oral health instead of comparison between an active agent and a control group, such as therapy and/or medication?

Yes = include this study

No = exclude this study

**C.2. Full-text Reviewing Checking List**

Guiding Screening Question: Does the study include data collection methods of collecting patient-reported surveys to measure oral health for care in quantitative research?

Yes = Included

No = Excluded

To answer ‘Yes’ to the above guiding question, the study must meet all following criteria:

1. Do we find a full text of this study?

Yes = move to the next criteria

No = exclude this study

1. Is this study in English?

Yes = move to the next criteria

No = exclude this study

1. Does this study have a self-reported and/or proxy-reported survey to assess oral health status? Oral health status is a measure of overall condition of oral cavity, including concepts, such as oral quality of life, oral health impact, and subject rating of oral health.

Yes = move to the next criteria

No = exclude this study

1. Is this measure of oral health the primary objective of this study, even though other measures of general health or other diseases are included?

Yes = move to the next criteria

No = exclude this study

1. Is it primarily a quantitative survey research study?

Yes = move to the next criteria

No = exclude this study

1. Does the survey have more than 3 questions related oral health?

Yes = move to the next criteria

No = exclude this study

1. Does this study collect primary data to analyze?

Yes = move to the next criteria

No = exclude this study

1. Does this study use legacy measures to assess the oral health status of a population rather than merely validating of legacy measures of oral health?

Yes = move to the next criteria

No = exclude this study

1. Does this study focus on the self-reported oral health instead of comparison between an active agent and a control group, such as therapy and/or medication?

Yes = move to the next criteria

No = exclude this study

1. Does this research describe sufficient information to ensure a complete understanding of the data collection methods?

Yes = include this study

No = exclude this study
