# Supplementary material for: Quantitative data collection approaches in subject-reported oral health research: a scoping review
Source: BMC Oral Health. 2022 Oct 3;22:435. doi: 10.1186/s12903-022-02399-5 (PMC9528129; doi:10.1186/s12903-022-02399-5)
Supplement: Supplementary file 5 — Additional file 5. Glossary of Terms. [file 12903_2022_2399_MOESM5_ESM.docx]

**Appendix E. Glossary of Terms**

| **Terms** | **Definition in This Paper** |
| --- | --- |
| Measures | Survey items to which the participant responds, including single-item self-reported oral health status, oral health related quality of life assessments, symptom checklists, and diverse survey questions on perceptions of oral health, oral pain, disease and treatment. |
| Method | A particular form of procedure for accomplishing or approaching something, especially a systematic or established one. In survey research, a quantitative method to collect information from a pool of respondents by asking multiple questions, the variables of interest are measured using self-reports and considerable attention is paid to the issue of sampling. |
| Methodological Approach | The approach taken to explore a research topic that determines the data gathering process. The approach of all papers selected for this scoping review is that of quantifying data and generalizing results from a sample of a target population following a structured data collection process, with objective analysis using statistical means. |
| Mode | The way of administering a survey, including face-to-face, mail, telephone, online and mobile data collection techniques. |
| Oral Health Functioning Measure | Questionnaires to access physical, psychological, and social functions. The sum or summarized scores can be used to represent the overall function condition. |
| Oral Health Impact Measure | Oral Health Impact Measure: Questionnaires to measure oral health impact. The sum or summarized scores can be used to represent  1. physical (e.g., being hard to eat due to pain in the mouth);  2. psychological (e.g., feeling stress due to pain in the mouth);  3. social (e.g., staying away from friends due to pain in the mouth)  impacts caused by functional limitations on oral health. |
| Oral Health Status | Health status of the teeth, gums, and the entire oral-facial system that allows for physical, psychological, and social normal life. |
| Perceived Oral Health Status and Functioning Measure | Questionnaires to measures perceived oral health status score, functional limitations, emotional and social well-being. The sum or summarized scores can be used to represent the overall function condition. |
| Proxy-rating OHS | Some other person (e.g., parent, or a caregiver) report the individual oral health status on behalf of the subject of interest. |
| Quantitative Method | Quantitative research involves the use of quantifiable, or numerical, data, such as using surveys and questionnaires. |
| Recruitment Method | The process of actively searching and including participants for the study. |
| Self-rating OHS | Subject reports the oral health status. |
| Technological Approach | Computerized modes, internet-supported devices and interactive web technology as recent innovations for survey questionnaire design, sampling and recruiting, communication with respondents and data collection. |
